# Oligonuclear Manganese Complexes with Multiple Redox Properties for High-Contrast Electrochromism

**DOI:** 10.3390/molecules30092054

**Published:** 2025-05-05

**Authors:** Yi-Ting Wu, Hao-Tian Deng, Li-Yi Zhang, Meng-Die Li, Feng-Rong Dai, Zhong-Ning Chen

**Affiliations:** 1 State Key Laboratory of Structural Chemistry, Fujian Institute of Research on the Structure of Matter, Chinese Academy of Sciences, Fuzhou 350002, China; wuyiting22@mails.ucas.ac.cn (Y.-T.W.); denghaotian@fjirsm.ac.cn (H.-T.D.); limengdie@fjirsm.ac.cn (M.-D.L.); 2 University of Chinese Academy of Sciences, Beijing 100049, China; 3 Fujian College, University of Chinese Academy of Sciences, Fuzhou 350002, China

**Keywords:** manganese complexes, mixed-valent compounds, redox, electrochromism, electrochromic device

## Abstract

This study is dedicated to the design of multiple redox-active oligonuclear manganese complexes supported with a bis(tetradentate) ligand (TPDP = 1,3-bis(bis(2-pyridinylmethyl)amino)-2-propanol) for high-contrast electrochromism based on the reversible redox process between Mn(II) (colorless) and Mn(III) (dark brown). Pentanuclear Mn_5_ complex **1** (colorless) was synthesized via a one-pot reaction of Mn^2+^ and TPDP, while tetranuclear Mn_4_ complex **2** (brown) was obtained through aerial oxidation of complex **1**. Mn_5_ complex **1** features a central MnCl_6_ unit connected to two Mn_2_(*μ*-TPDP) fragments through *μ*_3_-Cl^−^ and *μ*-Cl^−^, whereas Mn_4_ complex **2** adopts a symmetric tetranuclear structure with two mixed-valence Mn_2_^II,III^(*μ*-TPDP)(*μ*-Cl) fragments that are further linked by *μ*-oxo. Electrochemical studies revealed multi-step reversible redox properties for both complexes, attributed to Mn^II^/Mn^III^ processes with significant electronic coupling (Δ*E*_1/2_ = 0.27–0.37 V) between Mn centers. Spectroelectrochemical analysis revealed dynamic optical modulation through the tunable d-d transition and ligand-to-metal charge transfer (LMCT) state through reversible multiple redox processes based on Mn(II) ⇆ Mn(III) interconversion. The fabricated electrochromic device (ECD) exhibited reversible and high optical contrast between the colored state (dark brown) and the bleaching state (colorless). The results highlight the potential of polynuclear manganese complexes as high-contrast electrochromic materials for next-generation smart windows and adaptive optical technologies.

## 1. Introduction

Polynuclear metal complexes, characterized by multiple metal centers linked with organic or inorganic ligands, have attracted significant attention in materials science due to their synergistic electronic interactions, structural diversities, and multifunctional properties [1,2,3,4,5,6,7,8,9,10,11,12,13]. Among them, polynuclear manganese complexes stand out for their unique redox dynamics, magnetic coupling effects, and tunable catalytic activities with great potential for applications in bioinorganic chemistry (e.g., mimicking the water-oxidizing center in photosynthesis), energy conversion (e.g., oxygen evolution catalysis), and molecular electronics (e.g., spintronic devices) [14,15,16,17,18,19,20,21,22]. It is known that the manganese complexes, such as manganese phthalocyanine derivatives, show remarkable potential in electrochromic application owing to the distinctive electronic structure and redox versatility of Mn ions [23,24,25]. Taking into consideration the unique properties of multi-electron redox chemistry and the efficient charge transport characteristic, polynuclear manganese complexes would emerge as promising candidates for electrochromic applications.

**Scheme 1:**
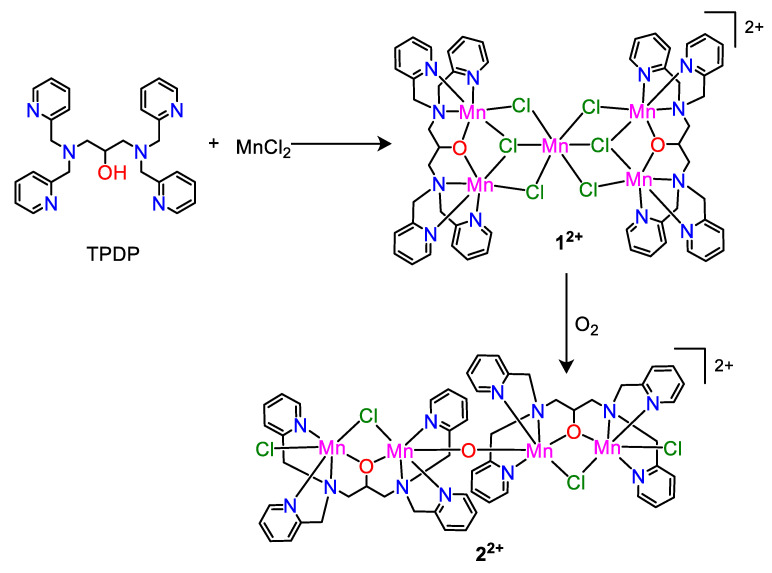
Synthetic routes to TPDP-based polynuclear manganese complexes **1^2+^** and **2^2+^**.

Electrochromic devices (ECDs), as dynamic systems capable of modulating optical properties via electric fields, hold significant application potential in smart windows, low-power displays, adaptive camouflage coatings, and energy-efficient buildings. Although conventional electrochromic materials, such as tungsten trioxide (WO_3_) and conducting polymers (e.g., PEDOT:PSS), have achieved commercial success, they still face limitations in color tunability, redox reversibility, and scalability. Polynuclear Mn complexes intrinsically satisfy criterion for electrochromic applications owing to the unique properties of multi-metal redox centers, multiple accessible oxidation states (II-IV), and ligand-field-tunable electronic structures. In this study, we focus on the designed synthesis and electrochromic performance of pentanuclear and tetranuclear manganese complexes (Scheme 1) with a heptadentate ligand (TPDP) as the chelating ligand. The TPDP ligand not only provides an easily deprotonated -OH group to promote the generation of the (*μ*_2_-O)M_2_ coordination model, but it also serves as an efficient chelating ligand to stabilize metal centers through dual N^N^N coordination modes. By bridging molecular engineering and functional materials science, this study aims to advance electrochromic technologies with enhanced efficiency, durability, and environmental compatibility.

## 2. Results and Discussion

### 2.1. Synthesis and Crystal Structure

Pentanuclear complex [Mn_5_(TPDP)_2_(*μ*_3_-Cl)_2_(*μ*-Cl)_4_](ClO_4_)_2_ (**1**(ClO_4_)_2_) was prepared by the reaction of the ligand TPDP and MnCl_2_ in a mixed solvent of CH_3_CN/CH_3_OH under an inert atmosphere at 85% yield as a colorless solid, implying that the formal oxidation states of the five Mn atoms were all +2 (valence II). When the CH_2_Cl_2_ solution of complex **1**(ClO_4_)_2_ was exposed in air, the colorless solution rapidly turned brown, thus leading to the isolation of tetranuclear complex [Mn_4_(TPDP)_2_(*μ*-O)(*μ*-Cl)_2_Cl_2_](ClO_4_)_2_ (**2**(ClO_4_)_2_) as a brown solid, which possesses a mixed valence state of Mn_2_^II,III^(*μ*-O)Mn_2_^II,III^. For complex **1**(ClO_4_)_2_ (vide infra), the low potential (*E*_1/2_(A) = 0.058 V vs. Fc^+^/Fc,) of the first oxidation wave, corresponding to the [Mn_2_^II,II^-Mn^II^Cl_6_-Mn_2_^II,II^]^2+^ ⇆ [Mn_2_^II,II^-Mn^III^Cl_6_-Mn_2_^II,II^]^3+^ process, prompts it to be oxidized by O_2_ into [Mn_2_^II,II^-Mn^III^Cl_6_-Mn_2_^II,II^]^3+^ species (**1**^3+^) with the simultaneous formation of an oxo (O^2−^) dianion. Since the Mn^III−^O^2−^ bond is much stronger than that of Mn^III−^Cl, Mn_5_ complex **1**(ClO_4_)_2_ was thus converted to mixed-valence Mn_4_^II,II,III,III^ complex **2**(ClO_4_)_2_. In fact, complex **1**(ClO_4_)_2_ can be viewed as consisting of two Mn_2_(TPDP)Cl_3_ units and one Mn^2+^, incorporating together through the linkage of six Cl^−^ in *μ*-Cl (four) and *μ*_3_-Cl (two) bonding modes. Once the central Mn^2+^ is oxidized to Mn^3+^ in air, the Mn^III−^Cl bond is disrupted with the formation of a Mn^III−^O^2−^ bond. As a result, two Mn_2_(TPDP)Cl_3_ units are linked together through a *μ*-O^2−^ dianion to produce complex **2**(ClO_4_)_2_.

**Figure 1:**
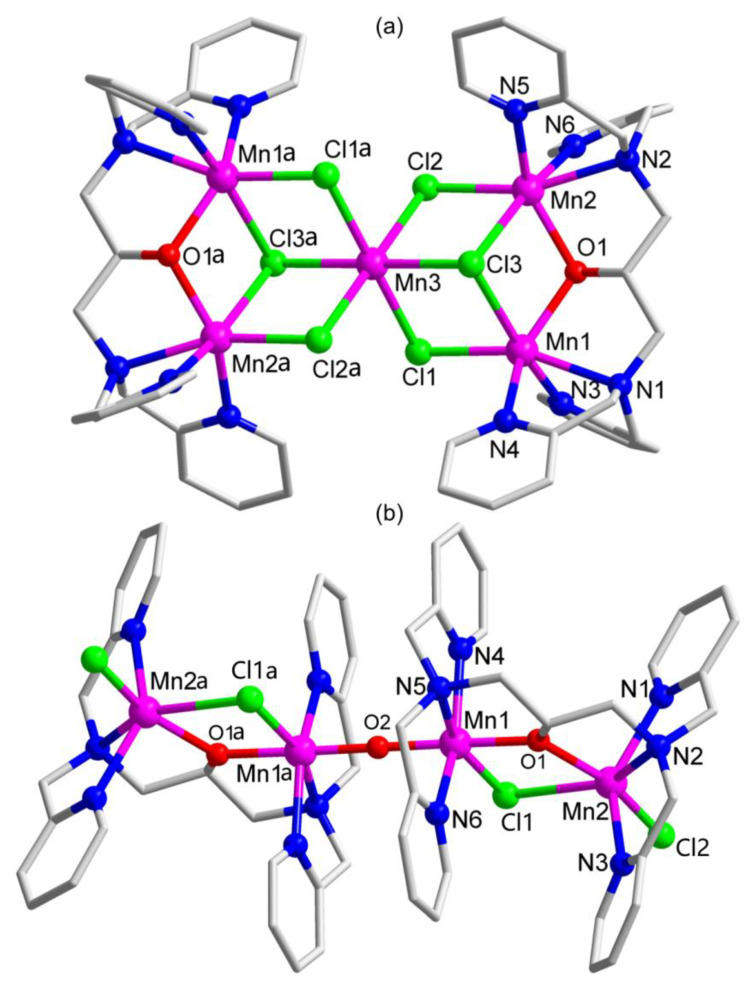
Perspective views of complex cations of **1^2+^** (**a**) and **2^2+^** (**b**) from X-ray crystallography. Color scheme: Mn, pink; Cl, green; O, red; C, gray; N, blue. The hydrogen atoms have been deleted for clarity.

The structures of complexes **1**(ClO_4_)_2_ and **2**(ClO_4_)_2_ were characterized by the single-crystal X-ray Diffraction (SC-XRD) analysis. As is shown in Figure 1a, the pentanuclear manganese cation **1**^2+^ is composed of two Mn_2_(TPDP) and one MnCl_6_ fragments put together through the Mn-Cl-Mn linkages. The TPDP is bound to two Mn^II^ atoms through the formation of six five-membered chelating rings, behaving as a bis(tetradentate) bridging–chelating ligand, where two Mn^II^ atoms are linked by a deprotonated hydroxyl. The bond angles around O1 are 109.0, 110.0, and 114.3°, indicating a typical sp^3^ hybridization of the hydroxyl O atom [26]. The octahedral coordination sphere of the TPDP-chelated Mn atom is built by one hydroxyl O, one tertiary amine N atom, two pyridine N atoms, one *μ*-Cl, and one *μ*_3_-Cl atom. Noticeably, the two Mn^II^ centers bridged by TPDP exhibit a highly distorted coordination environment with the bond angles around the Mn^II^ center varying from 73.4° (N4-Mn1-N1) to 117.9° (O1-Mn1-Cl1). The bond lengths are 2.080−2.089 Å for Mn−O, 2.231−2.363 Å for Mn−N, 2.239−2.248 Å for Mn−(*μ*_3_-Cl), and 2.779−2.787 Å for Mn−(*μ*-Cl). The central Mn center bound to six Cl^−^ atoms adopts a slightly distorted octahedral coordination geometry with Mn−Cl bond lengths of 2.530–2.566 Å and Cl−Mn−Cl bond angles of 86.95–93.05°. The Mn···Mn distance across the bridging hydroxyl and *μ*_3_-Cl is 3.503 Å (Mn1−Mn2), while those across *μ*-Cl and *μ*_3_-Cl are 3.772 Å (Mn1−Mn3) and 3.781 Å (Mn2−Mn3), suggesting a delocalized electronic structure to exhibit multiple redox behaviors.

Complex cation **2^2+^** (Figure 1b) consists of two symmetric Mn_2_ units linked by a *μ*-O^2−^ atom to form a quasi-linear tetranuclear cluster. The *μ*-O^2−^ atom locates at the inversion center to give the Mn1-O2-Mn1a angle of 180°. The quite short Mn1–O2 length suggests that Mn1 is typical of valence III [27,28]. The bridging pathways of Mn1-O1-Mn2 (Mn^III^-O-Mn^II^) and Mn1-Cl1-Mn2 (Mn^III^-Cl-Mn^II^) are severely asymmetric, where the much shorter Mn1–O1 (1.907(2) Å) bond than that of Mn2–O1 (2.129(2) Å) or the much shorter Mn1–Cl1 (2.4361(9) Å) distance than that of Mn2–Cl1 (2.6821(9) Å) further confirms the valence III characteristic for Mn1, in striking contrast to the character of valence II for Mn2. Mn1 (valence III) or Mn2 (valence II) exhibits distorted octahedral coordination geometry composed of ClN_3_O_2_ donors for Mn1 and Cl_2_N_3_O donors for Mn2. Owing to the valence III character of Mn1, the Mn1–N_amine_ (2.245(3) Å) and Mn1–N_py_ (2.127(3)–2.147(3) Å) bonds are much stronger than the corresponding Mn2–N_amine_ (2.433(3) Å) and Mn2–N_py_ (2.218(3)–2.224(3) Å) bonds. The Mn1···Mn1a distance through the bridging *μ*-O^2−^ atom is 3.5245(6) Å, which is a little longer than that of Mn1···Mn2 (3.5089(6) Å) across the bridging *μ*-Cl^−^ atom.

### 2.2. Electrochemical Characterization

**Figure 2:**
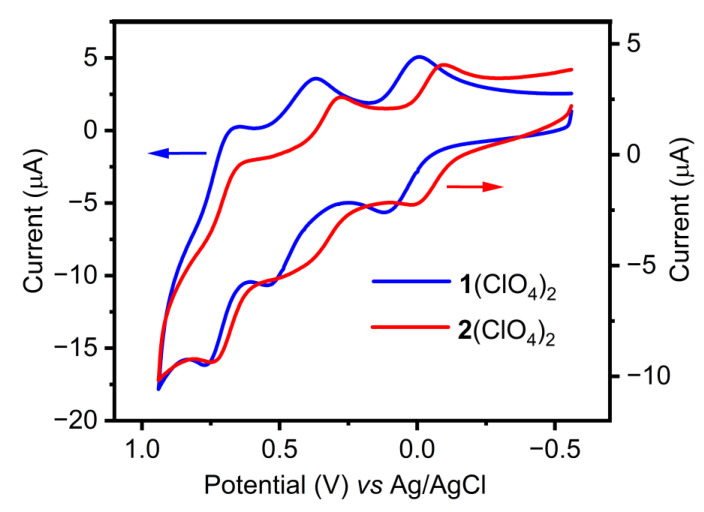
Cyclic voltammograms for of complexes **1**(ClO_4_)_2_ (blue) and **2**(ClO_4_)_2_ (red) in 0.1 M CH_3_CN solutions of (Bu_4_N)(ClO_4_). The scan rates was 100 mV/s.

The redox properties of complexes **1**(ClO_4_)_2_ and **2**(ClO_4_)_2_ were examined by cyclic voltammetry (CV) in acetonitrile solutions. As is shown in Figure 2, complex **1**(ClO_4_)_2_ exhibits three pair of distinct reversible redox waves in the potential range of −0.50 to +1.00 V vs. Fc/Fc^+^ (Table 1). The wave at +0.058 V (A) is attributed to the one-electron redox process of Mn^II^/Mn^III^ at the central Mn atom. The wave at +0.45 V (B) is ascribable to the superposition of two one-electron (corresponding to two-electron) oxidation processes of two separated TPDP-linked Mn_2_ units from Mn^II^Mn^II^ to Mn^III^Mn^II^. Another wave at +0.72 V (C) corresponds to the second double one-electron redox processes upon the further oxidation of an individual TPDP-linked Mn_2_ unit from Mn^III^Mn^II^ to Mn^III^Mn^III^. As a result, the redox chemistry of **1**(ClO_4_)_2_ is summarized as [Mn_2_^II,II^-Mn^II^-Mn_2_^II,II^]^2+^ (**1^2+^**) ⇆ [Mn_2_^II,II^-Mn^III^-Mn_2_^II,II^]^3+^ (**1^3+^**, *E*_1/2_(A) = 0.058 V) ⇆ [Mn_2_^III,II^-Mn^III^-Mn_2_^III,II^]^5+^ (**1^5+^**, *E*_1/2_(B) = 0.45 V) ⇆ [Mn_2_^III,III^-Mn^III^-Mn_2_^III,III^]^7+^ (**1^7+^**, *E*_1/2_(C) = 0.72 V). For each TPDP-linked Mn_2_ unit, the stepwise one-electron redox processes are Mn^II^Mn^II^ ⇆ Mn^III^Mn^II^ (*E*_1/2_(B) = 0.45 V) ⇆ Mn^III^Mn^III^ (*E*_1/2_(C) = 0.72 V) with a potential difference (Δ*E*_1/2_ = *E*_1/2_(C) − *E*_1/2_(B)) of 0.27 V, which is comparable to the corresponding value of other polynuclear manganese complexes [28,29,30] and demonstrates remarkable electronic communication between two Mn_2_ units mediated through bridging the TPDP ligand. Moreover, complex **1**(ClO_4_)_2_ showed excellent electrochemical stability over 5000 repeated CV cycles at the scan rate of 100 mV s^−1^ without an appreciable change in the CV curve.

As is depicted in Figure 2, complex **2**(ClO_4_)_2_ displays three pairs of reversible redox waves in the range of −0.50 to +1.00 V. The redox waves at −0.040 V (A) and +0.33 V (B) are most probably ascribable to stepwise one-electron redox processes of the two Mn^II^ ions connected by the *μ*_2_-O^2−^, that is, Mn^II^Mn^II^−O−Mn^II^Mn^II^/Mn^II^Mn^III^−O−Mn^II^Mn^II^ (*E*_1/2_(A) = −0.040 V) and Mn^II^Mn^III^−O−Mn^II^Mn^II^/Mn^II^Mn^III^−O−Mn^III^Mn^II^ (*E*_1/2_(B) = +0.33 V) with the potential difference (Δ*E*_1/2_ = *E*_1/2_(B) − *E*_1/2_(A)) of +0.37 V, implying significant electronic communication through the Mn−O^2−^−Mn bridging pathway [29]. The third redox wave at +0.69 V (C) most likely arises from concurrent double one-electron oxidation of two Mn^II^Mn^III^−O−Mn^III^Mn^II^ to Mn^III^Mn^III^−O−Mn^III^Mn^III^ units. Thus, the redox processes of **2**(ClO_4_)_2_ are described as [Mn^II^Mn^II^−O−Mn^II^Mn^II^] ⇆ [Mn^II^Mn^III^−O−Mn^II^Mn^II^]^+^ (*E*_1/2_(A) = −0.040 V) ⇆ [Mn^II^Mn^III^−O−Mn^III^Mn^II^]^2+^ (*E*_1/2_(B) = 0.33 V) ⇆ [Mn^III^Mn^III^−O−Mn^III^Mn^III^]^4+^ (*E*_1/2_(C) = 0.69 V).

### 2.3. Spectroelectrochemical Study

**Figure 3:**
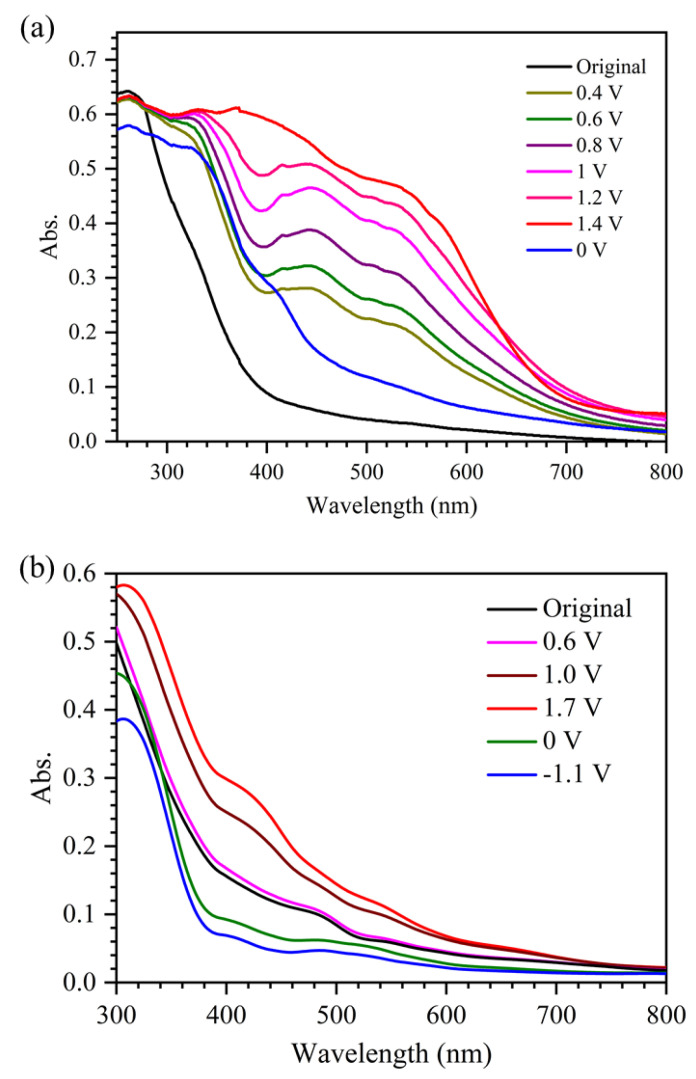
In-situ UV–Vis absorption spectra of complexes **1**(ClO_4_)_2_ (**a**) and **2**(ClO_4_)_2_ (**b**) in DMF at different external voltages.

Encouraged by the intriguing multiple redox characteristics, we next examined the spectroelectrochemical properties through the combination of an electrochemical workstation and UV–Vis spectrometer. A three-electrode system was installed in a quartz cuvette using the platinum plates as the working electrode and counter electrode, and Ag/AgCl as the reference electrode. The electrolysis of complexes **1**(ClO_4_)_2_ and **1**(ClO_4_)_2_ was performed using cyclic voltammetry with a scan rate of 10 mV/s. The absorption spectrum of complex **1**(ClO_4_)_2_ displays only a strong absorption band centered at 260 nm with a shoulder at the range of 300−370 nm due to the π → π* and n → π* transitions of TPDP (Figure S1 in Supplementary Materials), giving rise to it being colorless in acetonitrile solution in the initial state. This is consistent with the absence of a metal-to-ligand charge transfer (MLCT) and d-d transition of Mn^II^, which is forbidden [31,32,33,34]. Upon electrolysis of the complex **1**(ClO_4_)_2_ (Figure 3a) solution at an external potential of 0.4 V, the absorption band of 300−370 nm remarkably increased, followed by the appearance of two new sets of broad absorption bands in the visible region centered at 440 and 530 nm, ascribable to the oxidation of complex **1^2+^** ([Mn_2_^II,II^-Mn^II^-Mn_2_^II,II^]^2+^) into **1^3+^** ([Mn_2_^II,II^-Mn^III^-Mn_2_^II,II^]^3+^). The rise in absorption bands in the visible region along with the color changes in solution are likely attributed to the d-d transition of the central Mn^III^ ion, Cl^−^ → Mn^III^ LMCT (ligand-to-metal charge transfer, Figure S2) [35], and Mn^II^ → Mn^III^ IVCT (intervalence charge transfer) transitions. With the gradual increase in the redox potential to 0.8 V, the absorption bands in the visible region became progressively enhanced with the further oxidation following complex **1^2+^** → **1^3+^** → **1^5+^**, accompanied by the color deepening in solution because of the occurrence of an additional TPDP → Mn^III^ LMCT transition (Figure S3). When the applied external voltage was increased to 1.4 V, the solution became dark brown and the absorption intensity in the range of 400−700 nm reached a maximum with the observation of a new absorption peak centered at around 385 nm. Conversely, once the external potential bias was reduced to 0 V, the intensity of absorption bands in the visible region between 400 and 700 nm decreased sharply, indicating the recovery of the original state of complex **1**(ClO_4_)_2_.

As is shown in Figure 3b, complex **2**(ClO_4_)_2_ exhibits an intense UV absorption band attributed to π → π* and n → π* transitions of the TPDP ligand, along with composite visible-region absorption bands ascribed to the d-d transitions within the Mn^III^ ions, LMCT of O^2−^ → Mn^III^, and IVCT transitions of Mn^II^ → Mn^III^, resulting in a brown–yellow solution in the initial state (Figure S5). With an increasing applied external voltage, the absorption intensities of between 300 and 600 nm significantly increased, likely due to enhanced LMCT transitions from the TPDP ligand to Mn^III^ centers, indicating the oxidation of complex **2^2+^** ([Mn^II^Mn^III^−O−Mn^III^Mn^II^]^2+^) to **2^4+^** ([Mn_2_^III,III^−O−Mn_2_^III,III^]^4+^) (Figure S6). At a potential of 1.7 V, the visible-region absorption intensities reached a maximum, and the solution turned dark brown. When the external potential bias was reduced to 0 V or −1.1 V, the absorption intensities decreased remarkably, reflecting the reduction to the complex **2** state ([Mn^II^Mn^II^−O−Mn^II^Mn^II^]). Thus, the spectroelectrochemical studies demonstrated good reversibility in the electrochemical redox processes of complexes **1**(ClO_4_)_2_ and **2**(ClO_4_)_2_.

### 2.4. Electrochromic Device

**Figure 4:**
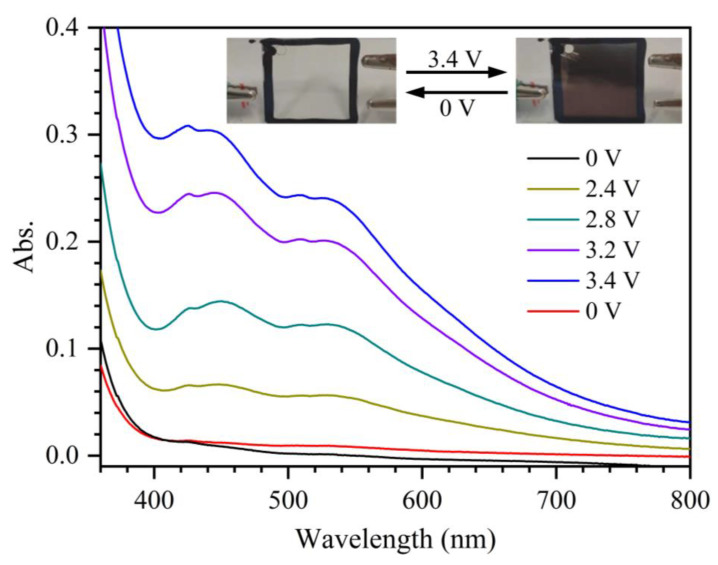
In-situ UV–Vis absorption spectra of electrochromic device based on complex **1**(ClO_4_)_2_ at different external voltages. The insets show the photographs of the color changes in electrochromic device after applying external potentials of 3.4 and 0 V.

To further understand the electrochromic properties of complexes **1**(ClO_4_)_2_ and **2**(ClO_4_)_2_, electrochromic devices with a sandwich structure of an ITO/PC solution of NBu_4_CF_3_SO_3_ (0.3 M) and complex **1**(ClO_4_)_2_ or **2**(ClO_4_)_2_ (0.01 M)/TiO_2_/ITO were fabricated in glove box using complex **1**(ClO_4_)_2_ or **2**(ClO_4_)_2_ as the electroactive material and a TiO_2_ thin film as the ion storage layer. As is shown in Figure 4, the electrochromic device appears colorless and transparent in its initial state with negligible absorption in the visible-light region. After applying an external potential at 2.4 V, the device began to darken and turned light brown, consistent with the appearance of two new broad absorption bands (centered at 450 and 530 nm) in the range of 400−800 nm. With the stepwise increase in the external potential, the color of the device gradually deepened, accompanied by a marked enhancement of the absorption bands. When the driving voltage was increased to 3.4 V, the device reached the saturation state and presented a dark-brown state. Moreover, the device can be controllably restored to its original colorless state (decolorated state) from the colored state by applying an external potential bias of 0 V, as evidenced by the vanishment of the absorption bands in the visible-light region (Figure 4). With stepping potentials between 0 and 3 V at an interval of 500 s, the transmittances of the device at 520 nm were collected, as shown in Figure S13. The coloration time (*t*_c_) and bleaching time (*t*_b_) of the device were calculated to be 360 and 120 s with an optical contrast of Δ*T* = 50% and a coloration efficiency (CE) of 44.6 cm^2^/C (Figure S14).

## 3. Materials and Methods

### 3.1. General Methods

Ligand TPDP was prepared according to the methods outlined in the literature [36]. The other chemicals were commercially available and used as received unless otherwise stated. The UV–Vis absorption spectra were measured on a UV2600i UV–Vis spectrophotometer (Shimadzu, Suzhou, China). Cyclic voltammograms (CVs) were recorded with an electrochemical analyzer (CHI 660E, Chenhua, Shanghai, China) in acetonitrile solutions containing 0.1 M (Bu_4_N)PF_6_ as the supporting electrolyte. Platinum, glassy graphite, and Ag/AgCl were used as the counter, working, and reference electrodes, respectively. The CVs were measured at a scan rate of 100 mV s^−1^. Spectroelectrochemical studies were conducted using a CHI 660E electrochemical workstation coupled with a UV2600i UV–Vis spectrometer, employing a three-electrode system in a quartz cuvette. The cuvette was placed in the sample holder of the UV–Vis spectrometer and connected to the electrochemical analyzer. The absorption spectra of the complexes under different applied voltages were monitored simultaneously during the electrochemical measurements.

### 3.2. Synthesis of ***1***(ClO_4_)_2_

A solution of manganese(II) chloride (0.095 g, 0.75 mmol), magnesium perchlorate (0.18 g, 0.50 mmol), and TPDP (0.23 g, 0.50 mmol) in mixed solvents of acetonitrile/methanol (*v*:*v* = 1:1, 20 mL) was stirred under a nitrogen atmosphere for 10 min. After the addition of diisopropylethylamine (0.55 mmol), the reaction mixture was heated to 80 °C with stirring for an hour. After reaction completion, the solvents were removed under vacuum. The solid was collected and washed with dichloromethane to give complex **1**(ClO_4_)_2_ as a colorless solid, with a yield of 85% (0.33 g). Elemental analysis calcd (%) for C_54_H_58_Cl_8_Mn_5_N_12_O_10_: C 40.70, H 3.67, N 10.55 and found C 40.61, H 3.82, N 10.48. HR-MS: *m*/*z* [M-(MnCl)-2(ClO_4_)]^+^ calcd 1303.0741 and found 1303.0879; [M-(MnCl_2_)-(ClO_4_)]^+^ calcd 1367.0537 and found 1367.0655. IR spectrum (KBr, cm^−1^): 1735 s, 1605 m, 1463 m, 1442 m, 1259 s, 1219 s, 1095 s, 1052 s, 766 m, 624 m, 523 m.

### 3.3. Synthesis of ***2***(ClO_4_)_2_

Compound **1**(ClO_4_)_2_ was dissolved in dichloromethane, and the solution was left exposed to the air. Brown crystals of complex **2**(ClO_4_)_2_ were obtained after 3 days, which were collected by filtration, and the yield was 92%. Elemental analysis calcd (%) for C_54_H_58_Cl_6_Mn_4_N_12_O_11_: C 43.72, H 3.94, N 11.33 and found C 43.16, H 3.88, N 10.56. HR-MS: *m*/*z* [M + 2OH + K-2(ClO_4_)]^+^ calcd 1357.1717 and found 1357.1715; [M-(OMn_2_TPDP)-2(ClO_4_)]^+^ calcd 633.0535 and found 633.0364. IR spectrum (KBr, cm^−1^): 1736 m, 1604 s, 1572 m, 1482 m, 1443 s, 1087 s, 1017 m, 921 m, 768 m, 731 m, 686 m, 621 m, 555 m, 417 m.

### 3.4. X-Ray Crystallography

X-ray single-crystal diffraction data were collected on a Bruker D8 Venture diffractometer (Bruker AXS GmbH, Karlsruhe, Germany) using an I*μ*S 3.0 microfocus source of Mo-Kα radiation (*λ* = 0.71073 Å) and a PHOTON II CPAD detector. Frames were integrated with the Bruker SAINT software package (V8.38A) using a SAINT algorithm. Data were corrected for absorption effects using the multi-scan method (SADABS). The structure was solved and refined using the Bruker SHELXTL (Version 2014) Software Package, a computer program for the automatic solution of crystal structures, and refined by the full-matrix least-squares method with ShelXle Version 4.8.6, a Qt graphical user interface for the SHELXL. CCDC 2432270-2432271 contain the supplementary crystallographic data for this paper. These data can be obtained free of charge from the Cambridge Crystallographic Data Centre via www.ccdc.cam.ac.uk/data_request/cif (accessed on 19th March 2025).

### 3.5. Fabrication of Electrochromic Devices

Indium-tin oxide (ITO)-coated glass slides (20 mm × 30 mm × 1.1 mm, *R*_s_ = 6 Ω/sq) were purchased from South China Xiangcheng Technology Co., Ltd. (Shenzhen China), which were cleaned by sequential washing with alkaline detergent, deionized water, ethanol, acetone, and isopropanol before used. The electrochromic devices (ECDs) were fabricated with a sandwich structure of the ITO/propylene carbonate (PC) solution (0.3 M NBu_4_CF_3_SO_3_ and 0.01 M **1**(ClO_4_)_2_/TiO_2_/ITO in a glove box. TiO_2_ was chosen as the ion storage layer and prepared using a sol–gel method. The TiO_2_ thin film was deposited on the ITO glass through pulling technology and annealed at 450 °C. The TiO_2_-coated ITO glass and another ITO-coated glass were joined together with butyl rubber placed along the whole perimeter. The active area of ECDs for testing was 20 × 20 mm^2^ with a thickness of 1 mm. A solution of NBu_4_CF_3_SO_3_ (0.3 M) and complex **1**(ClO_4_)_2_ (0.01 M) in PC was injected into the internal cavity by a syringe. The device was then sealed with photocurable adhesive and used for subsequent characterization.

### 3.6. Characterization of Electrochromic Devices

The device was directly placed in the sample holder of a UV–Vis spectrometer and connected to the electrochemical analyzer using a two-electrode configuration. Cyclic voltammetry of devices was conducted at a scan rate of 10 mV/s. The absorption or transmittance spectra of devices under different applied voltages were monitored simultaneously during the electrochemical measurements.

## 4. Conclusions

We demonstrated two novel polynuclear manganese complexes derived from a heptadentate TPDP ligand. Both exhibited multi-step reversible Mn^II^/Mn^III^ redox transitions owing to strong electronic coupling (Δ*E*_1/2_ = 0.27–0.37 V) between the Mn centers. Spectroelectrochemical studies revealed dynamic optical modulation through redox-triggered d-d, LMCT, and IVCT transitions, enabling reversible switching between colorless and dark-brown states. The corresponding electrochromic device (ECD) displayed reversible and high-contrast color changes, underscoring the potential of structurally tunable polynuclear manganese systems for advanced smart window technologies and adaptive optical applications. These findings establish a pathway for designing redox-versatile electrochromic materials with enhanced performance.

## Figures and Tables

**Table 1:** Electrochemical data of complexes **1**(ClO_4_)_2_ (blue) and **2**(ClO_4_)_2_.

	*E*_1/2_(A)	*E*_1/2_(B)	*E*_1/2_(C)
**1**(ClO_4_)_2_	0.058 ([Mn_2_^II,II^-Mn^II^-Mn_2_^II,II^]/ [Mn_2_^II,II^-Mn^III^-Mn_2_^II,II^])	0.45 ([Mn_2_^II,II^-Mn^III^-Mn_2_^II,II^]/ [Mn_2_^III,II^-Mn^III^-Mn_2_^III,II^])	0.72 ([Mn_2_^III,II^-Mn^III^-Mn_2_^III,II^]/ [Mn_2_^III,III^-Mn^III^-Mn_2_^III,III^])
**2**(ClO_4_)_2_	−0.040 [Mn^II^Mn^II^−O−Mn^II^Mn^II^]/ [Mn^II^Mn^III^−O−Mn^II^Mn^II^]	0.33 [Mn^II^Mn^III^−O−Mn^II^Mn^II^]/ [Mn^II^Mn^III^−O−Mn^III^Mn^II^]	0.69 [Mn^II^Mn^III^−O−Mn^III^Mn^II^]/ [Mn^III^Mn^III^−O−Mn^III^Mn^III^]

## Data Availability

Dataset available upon request from the authors.

## Supplementary Materials

The following supporting information can be downloaded at: https://www.mdpi.com/article/10.3390/molecules30092054/s1. Computational Method [37,38,39,40,41,42,43,44,45]; Figure S1: Density of states (DOS) and HOMO and LUMO of **1**^2+^ by B3LYP functional; Figure S2: Density of states (DOS) and HOMO and LUMO of **1**^3+^ by B3LYP functional; Figure S3: Density of states (DOS) and HOMO and LUMO of **1**^5+^ by B3LYP functional; Figure S4: Density of states (DOS) and HOMO and LUMO of **2** by B3LYP functional; Figure S5: Density of states (DOS) and HOMO and LUMO of **2**^2+^ by B3LYP functional; Figure S6: Density of states (DOS) and HOMO and LUMO of **2**^4+^ by B3LYP functional; Figure S7: The calculated absorption spectrum for **1**^+^ by B3LYP functional; Figure S8: The calculated absorption spectrum for **1**^3+^ by B3LYP functional; Figure S9: The calculated absorption spectrum for **1**^5+^ by B3LYP functional; Figure S10: The calculated absorption spectrum for **2** by B3LYP functional; Figure S11: The calculated absorption spectrum for **2**^2+^ by B3LYP functional; Figure S12: The calculated absorption spectrum for **2**^4+^ by B3LYP functional; Figure S13: Electrochromic switching times and stability of electrochromic device based on complex **1**(ClO_4_)_2_; Figure S14: Plot of the optical density (ΔOD) versus the charge density (C/cm^2^) of electrochromic device based on complex **1**(ClO_4_)_2_.

## Abbreviations

The following abbreviations are used in this manuscript:

TPDP	1,3-bis(bis(2-pyridinylmethyl)amino)-2-propanol
LMCT	Ligand-to-metal charge transfer
ECD	Electrochromic device
ITO	Indium tin oxide
CV	Cyclic voltammetry
MLCT	Metal-to-ligand charge transfer
IVCT	Intervalence charge transfer
