# Supplementary material for: Oligonuclear Manganese Complexes with Multiple Redox Properties for High-Contrast Electrochromism
Source: Molecules. 2025 May 5;30(9):2054. doi: 10.3390/molecules30092054 (PMC12073507; doi:10.3390/molecules30092054)
Supplement: Supplementary file 1 [file molecules-30-02054-s001.zip › molecules-3594512-supplementary.pdf]

Supporting Information

Oligonuclear Manganese Complexes with Multiple Redox Properties for High Contrast Electrochromism

Yi-Ting Wu[1,2], Hao-Tian Deng[1,2,3], Li-Yi Zhang[1,*], Meng-Die Li[1,2], Feng-Rong Dai[1,2,*], Zhong-Ning Chen[1,2,*]

[1] State Key Laboratory of Structural Chemistry, Fujian Institute of Research on the Structure of Matter, Chinese Academy of Sciences, Fuzhou, Fujian 350002, China; wuyiting22@mails.ucas.ac.cn (Y.-T.W.); denghaotian@fjirsm.ac.cn (H.-T.D.); limengdie@fjirsm.ac.cn (M.-D.L.)

[2] University of Chinese Academy of Sciences, Beijing 100049, China

[3] Fujian College, University of Chinese Academy of Sciences, Fuzhou 350002, China

* Correspondence: zhangliyi@fjirsm.ac.cn (L.-Y.Z.); dfr@fjirsm.ac.cn (F.-R.D.); czn@fjirsm.ac.cn (Z.-N.C.)

Computational Method. The theoretical calculations were implemented by using Gaussian 16 program package.[1] The geometrical structures of $\mathbf{1}^{2+}$, $\mathbf{1}^{3+}$, $\mathbf{1}^{5+}$, **2**, $\mathbf{2}^{2+}$, and $\mathbf{2}^{4+}$ in the ground states were firstly optimized by unrestricted density functional theory (DFT) method with the functional B3LYP[2-3] considering the unpaired electrons in Mn atoms. Then, in order to analyze the spectroscopic properties, 60 singlet excited-states were calculated, respectively, based on the optimized structures in the ground states to determine the vertical excitation energies by time-dependent density functional theory (TD-DFT)[4,5] with the same functional used in the optimization process. In the calculation of excited states, the polarizable continuum model method (PCM)[6,7] with acetonitrile for $\mathbf{1}^{2+}$, $\mathbf{1}^{3+}$, $\mathbf{1}^{5+}$ and N,N-Dimethylformamide for **2**, $\mathbf{2}^{2+}$, $\mathbf{2}^{4+}$as solvent was employed. In these calculations, the Stuttgart-Dresden (SDD)[8] basis set and the effective core potentials (ECPs) were used to describe the Mn atoms, while other non-metal atoms of Cl, O, N, C and H were described by the all-electron basis set of 6-31G**. Visualization of the orbitals (isovalue = 0.02) plots were performed by GaussView. The density-of-states were analyzed in Multiwfn 3.3.7 program.[9]

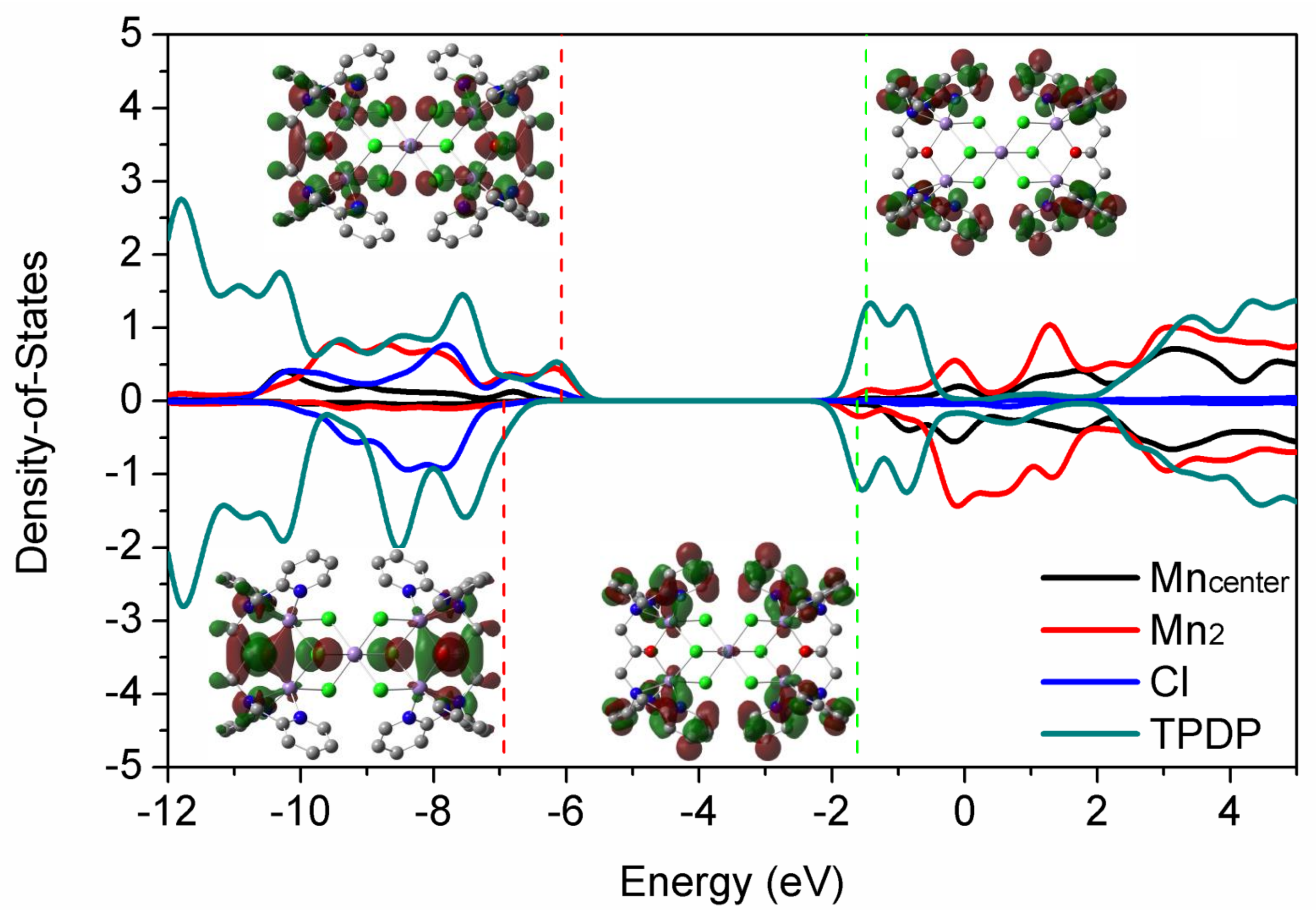

Figure S1. Density of states (DOS) and HOMO and LUMO of $\mathbf{1}^{2+}$ by B3LYP functional.

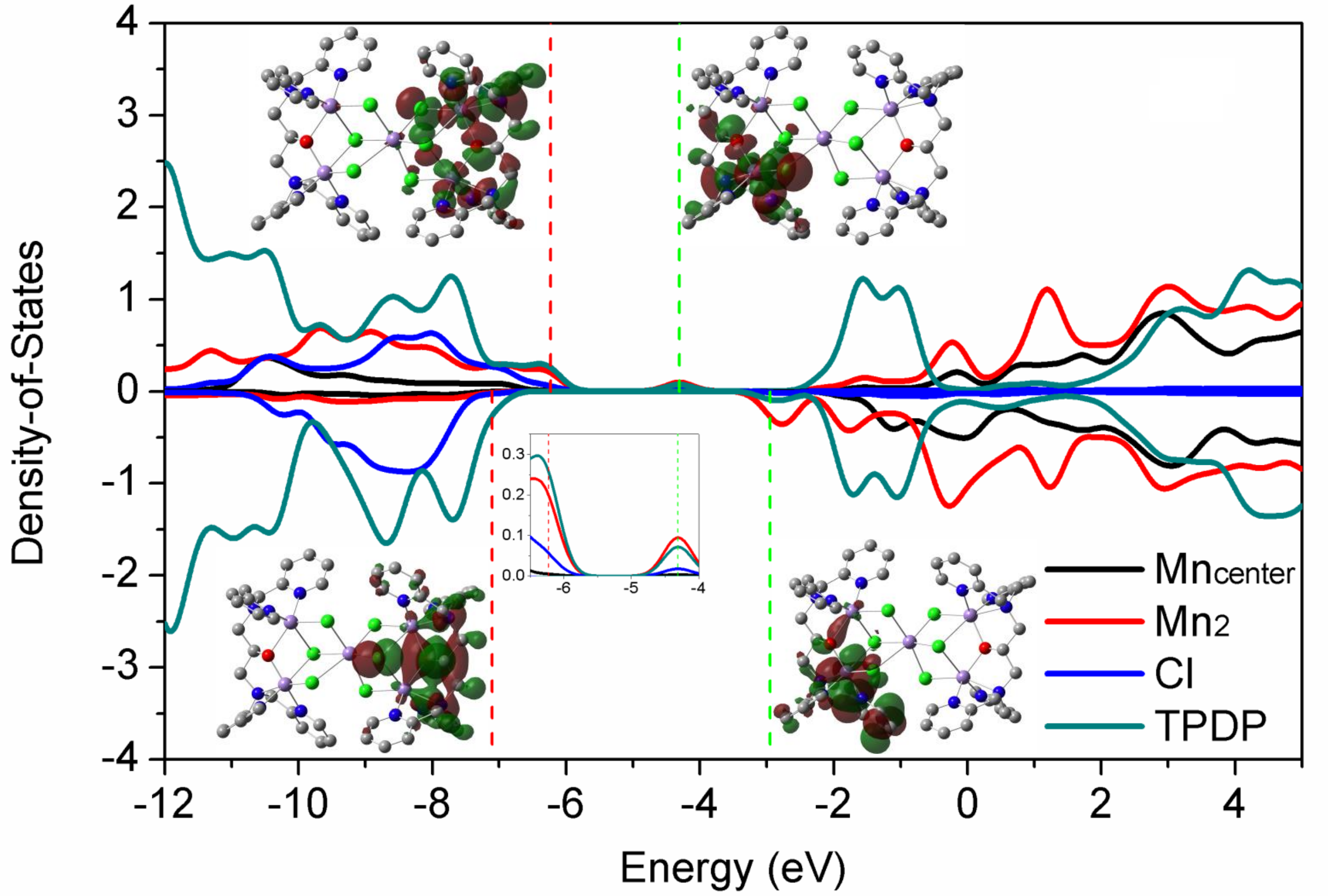

Figure S2. Density of states (DOS) and HOMO and LUMO of **1**$^{3+}$ by B3LYP functional.

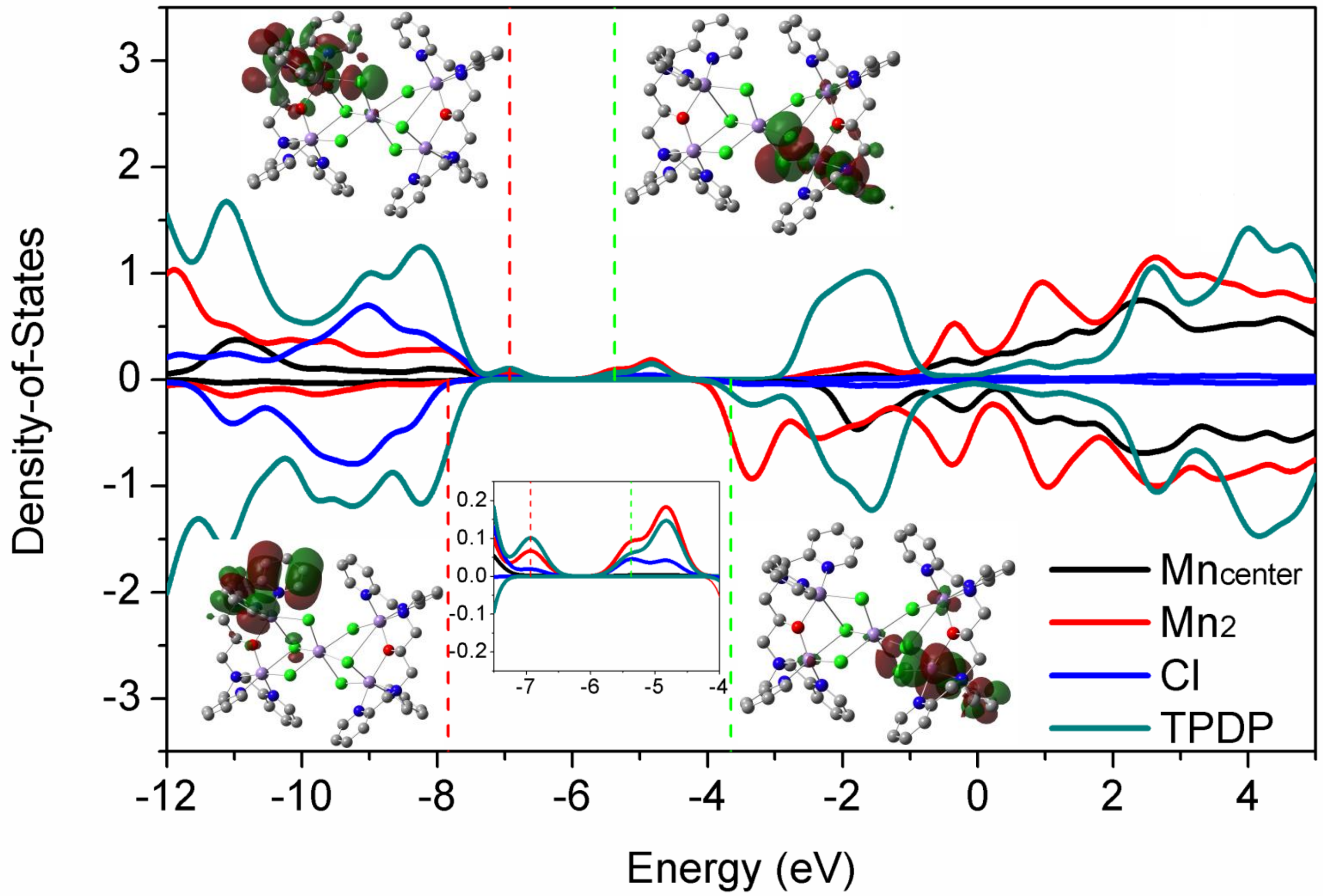

Figure S3. Density of states (DOS) and HOMO and LUMO of **1**$^{5+}$ by B3LYP functional.

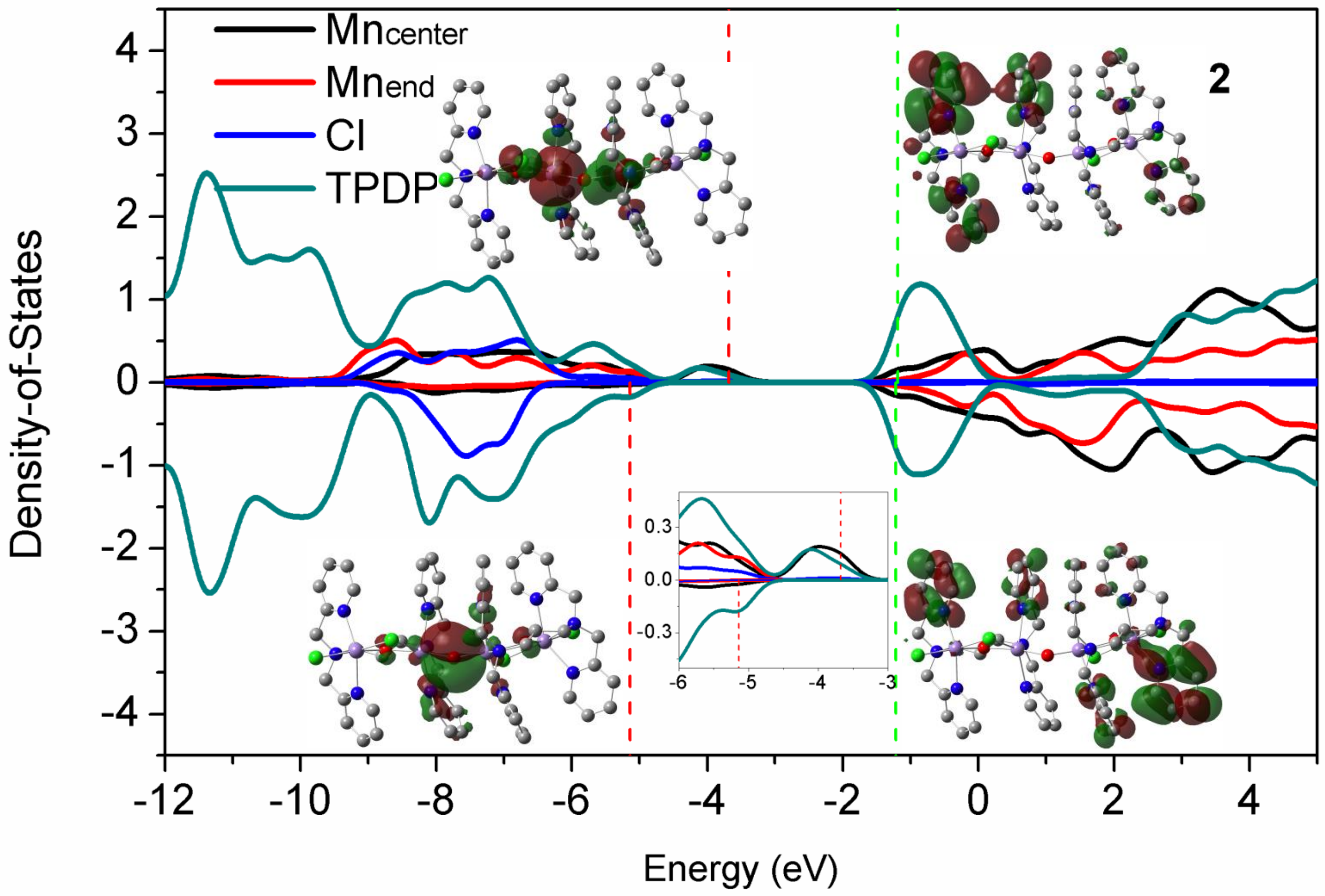

Figure S4. Density of states (DOS) and HOMO and LUMO of **2** by B3LYP functional.

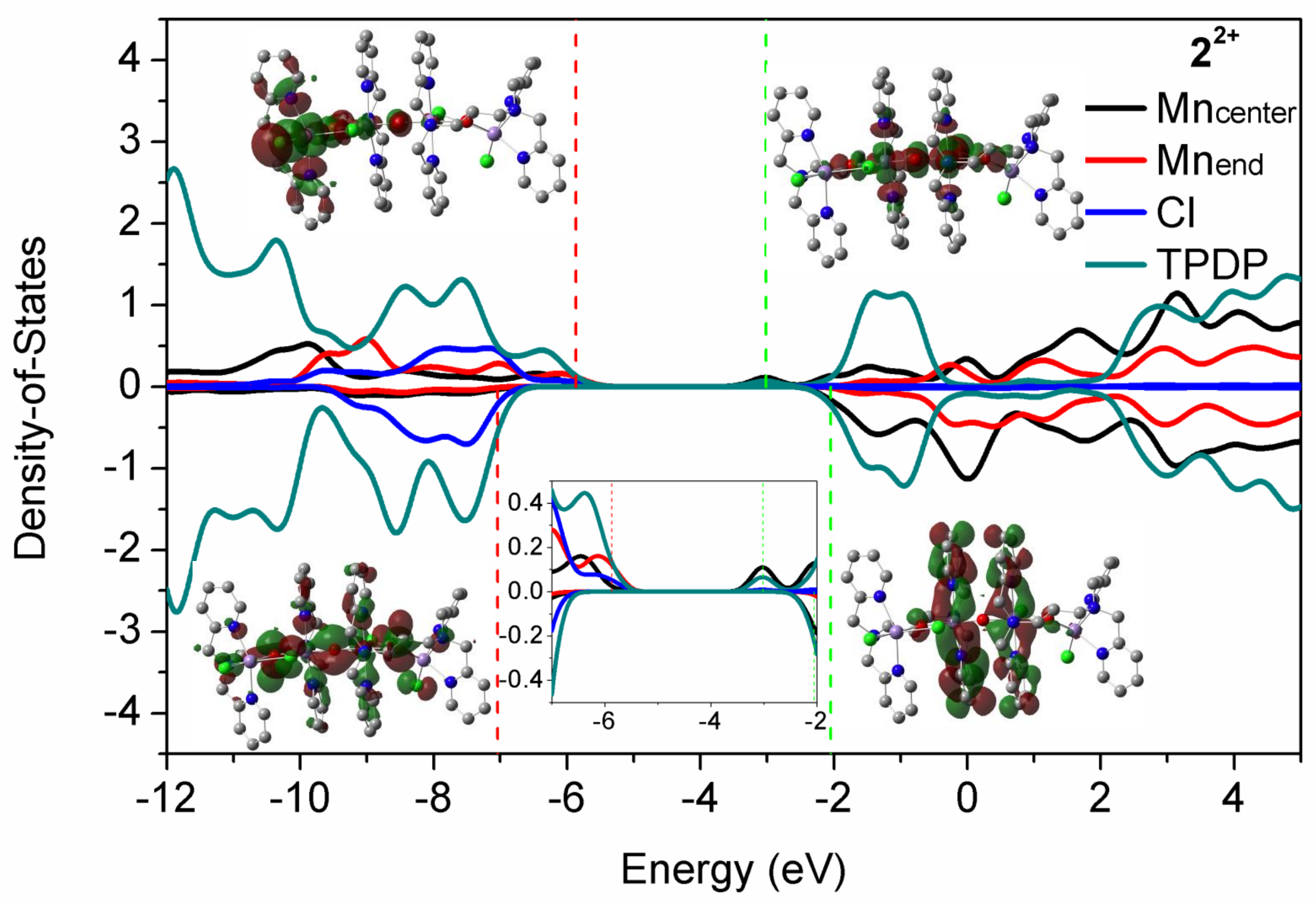

Figure S5. Density of states (DOS) and HOMO and LUMO of **2**$^{2+}$ by B3LYP functional.

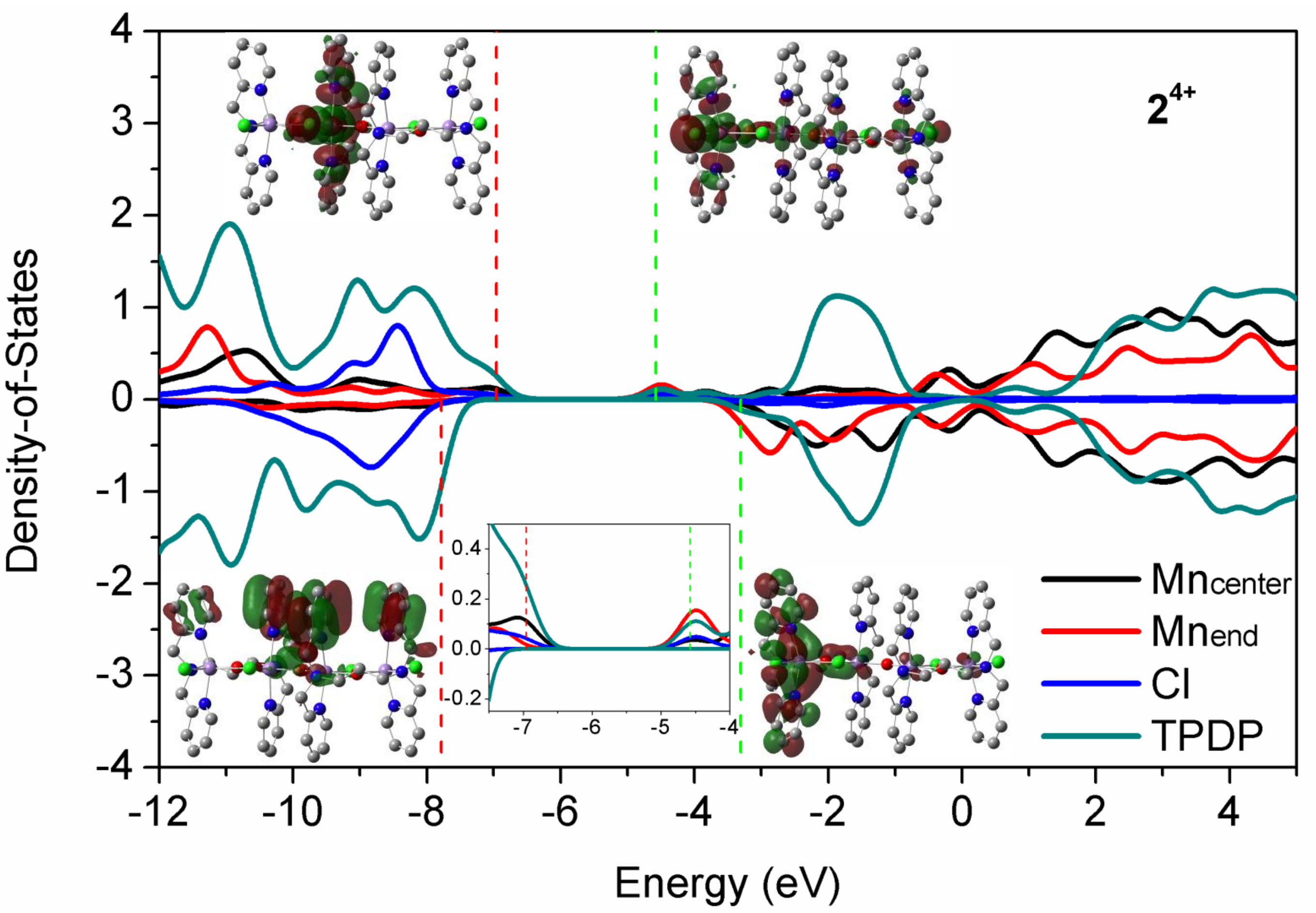

Figure S6. Density of states (DOS) and HOMO and LUMO of **2**$^{4+}$ by B3LYP functional.

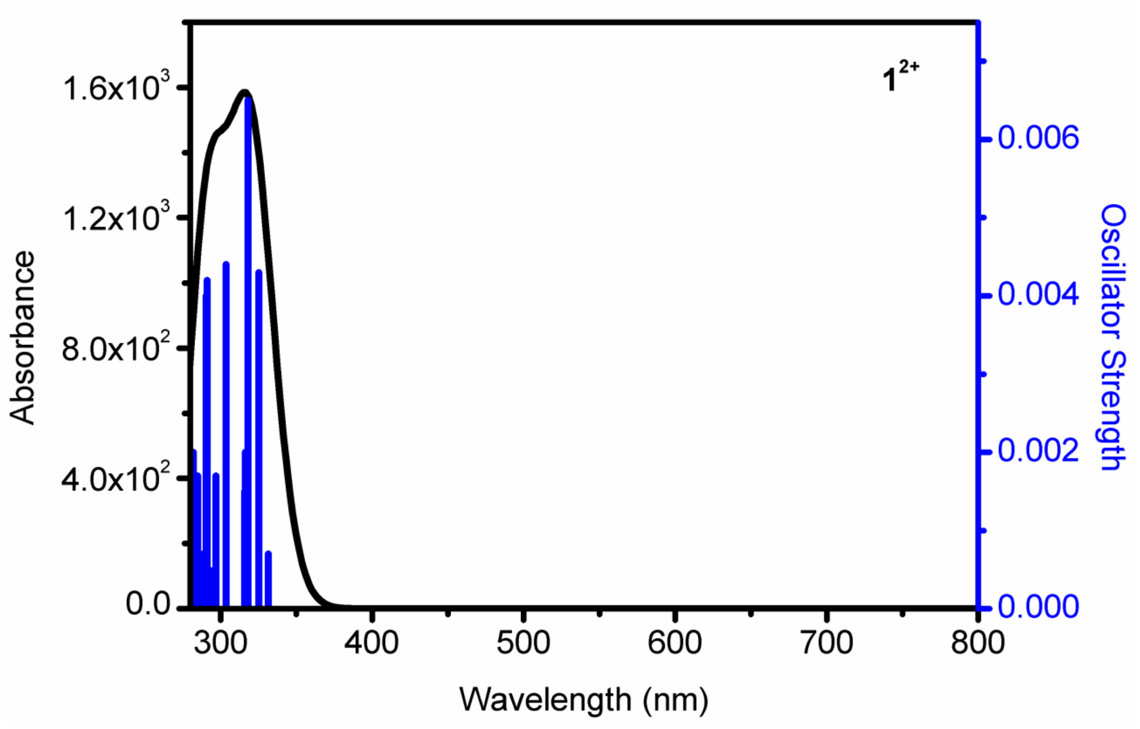

Figure S7. The calculated absorption spectrum for $\mathbf{1}^{+}$ by B3LYP functional.

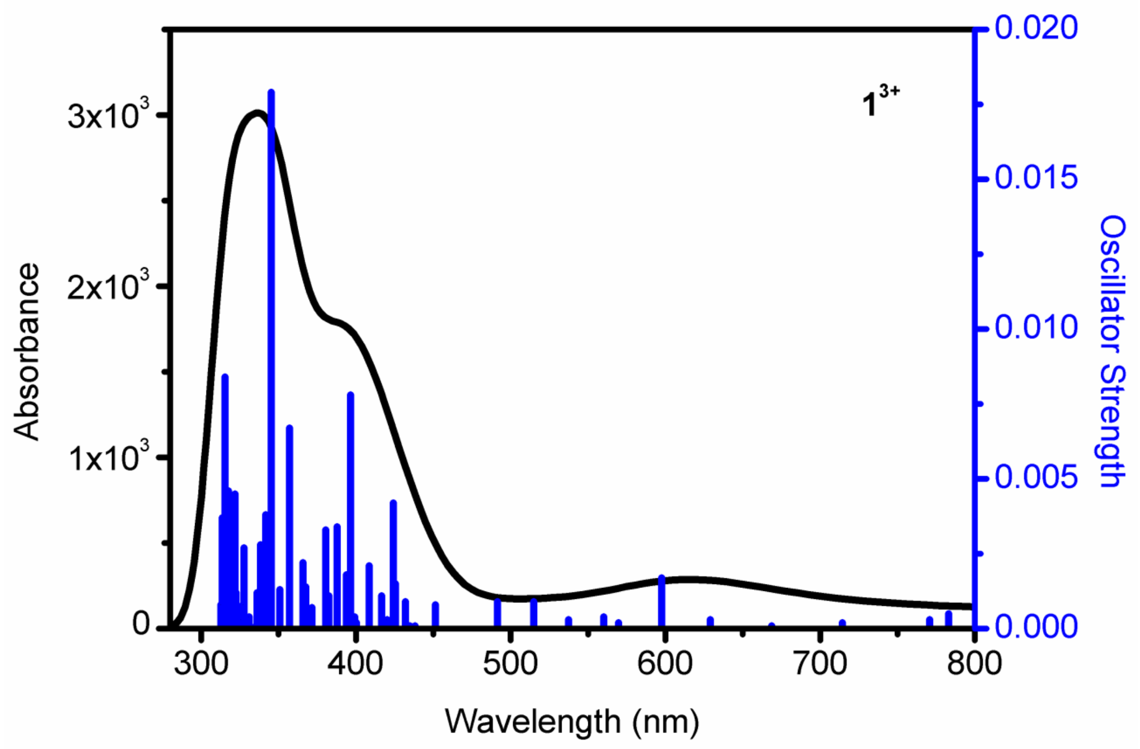

Figure S8. The calculated absorption spectrum for $\mathbf{1}^{3+}$ by B3LYP functional.

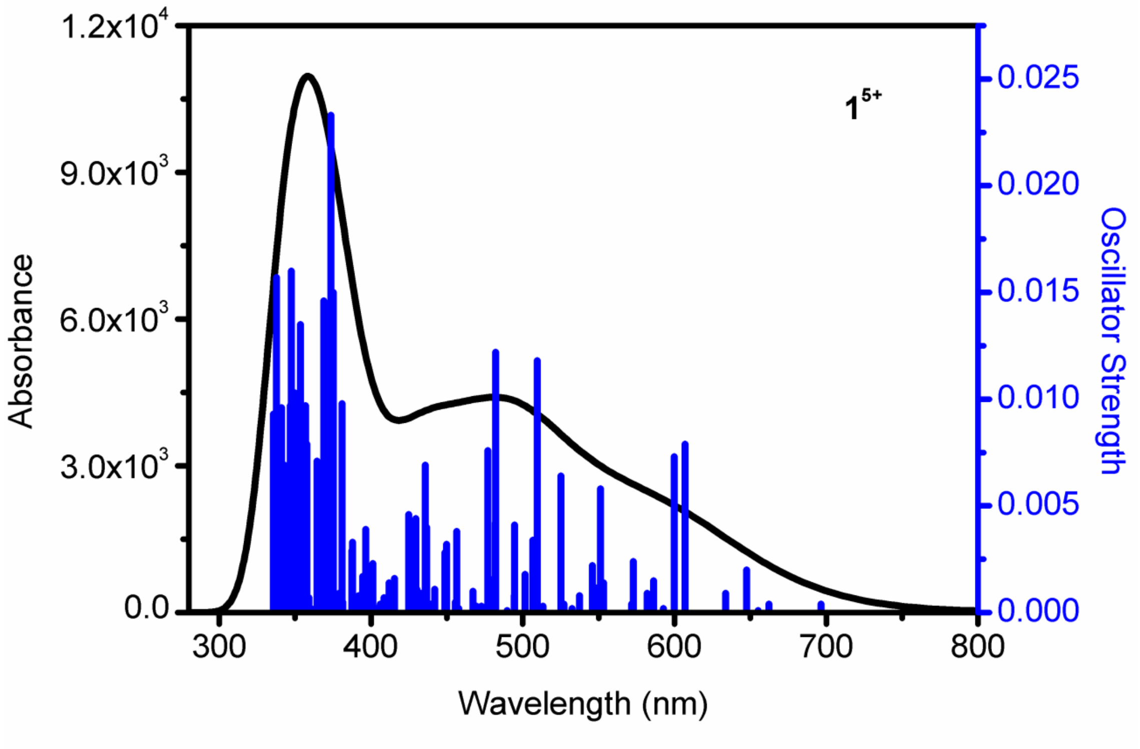

Figure S9. The calculated absorption spectrum for **1**$^{5+}$ by B3LYP functional.

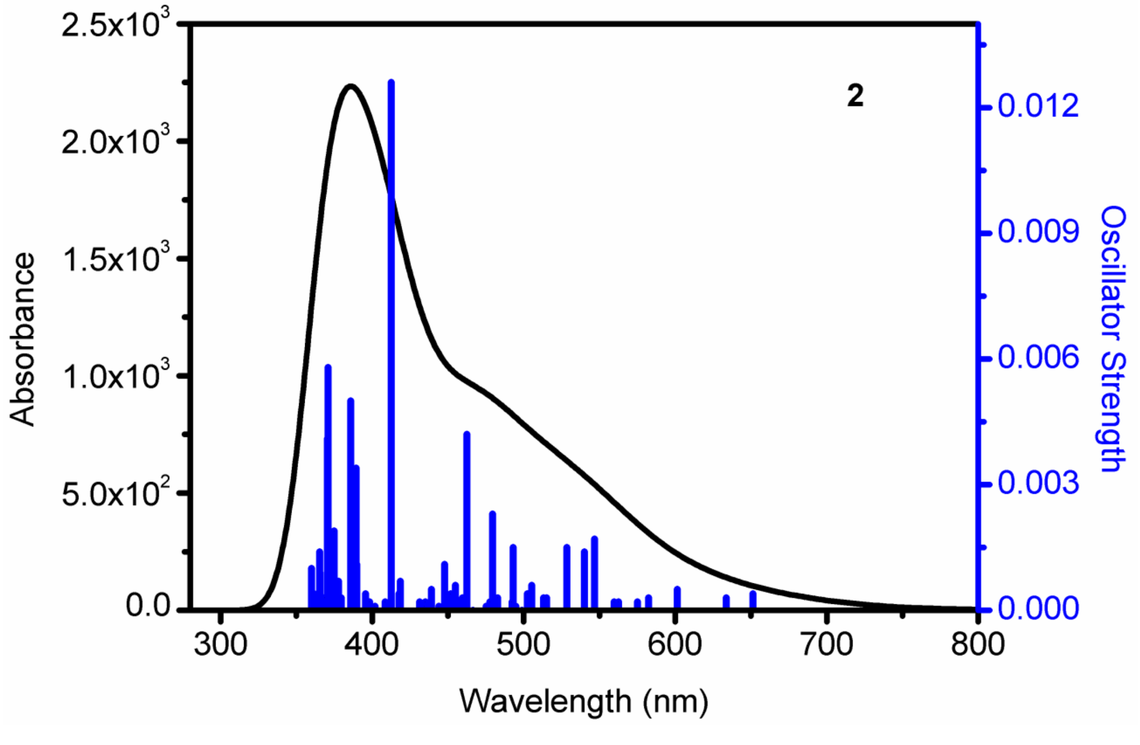

Figure S10. The calculated absorption spectrum for **2** by B3LYP functional.

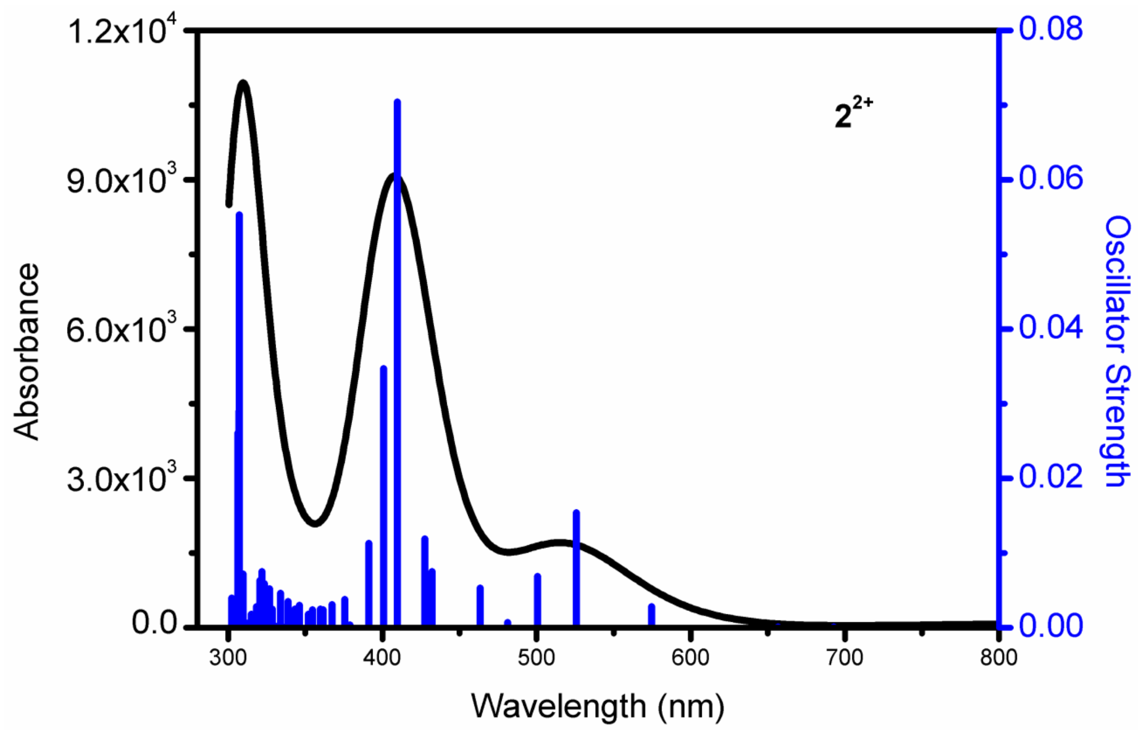

Figure S11. The calculated absorption spectrum for **2**$^{2+}$ by B3LYP functional.

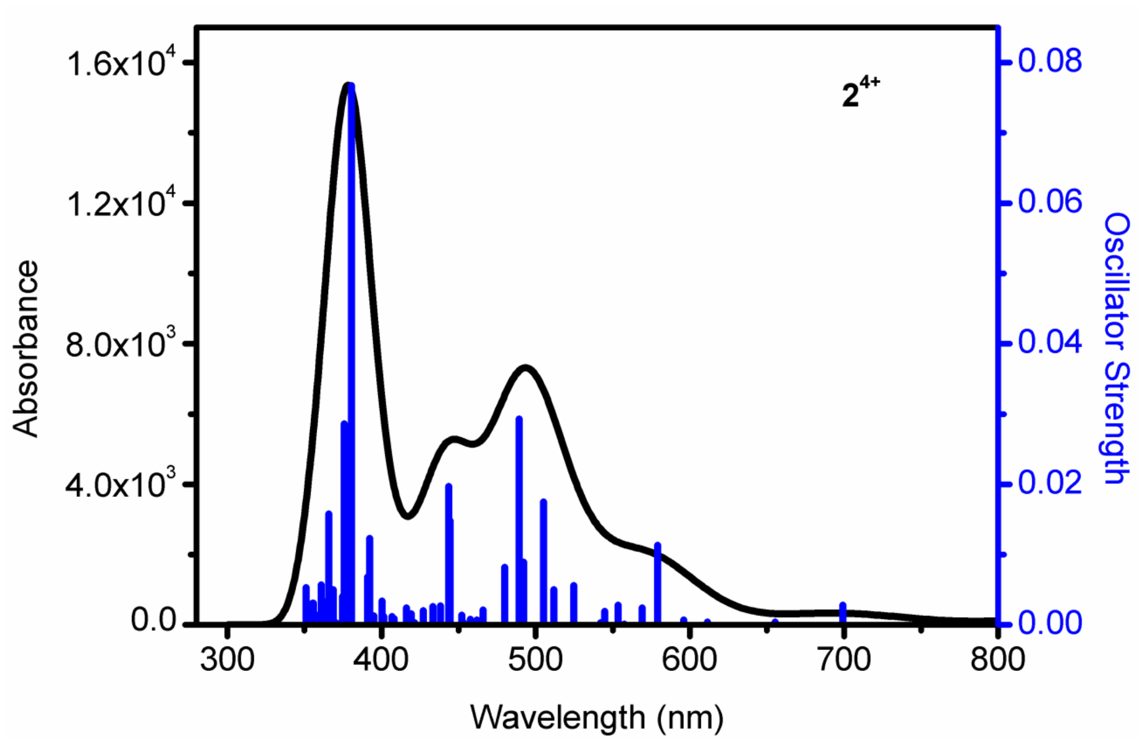

Figure S12. The calculated absorption spectrum for **2**$^{4+}$ by B3LYP functional.

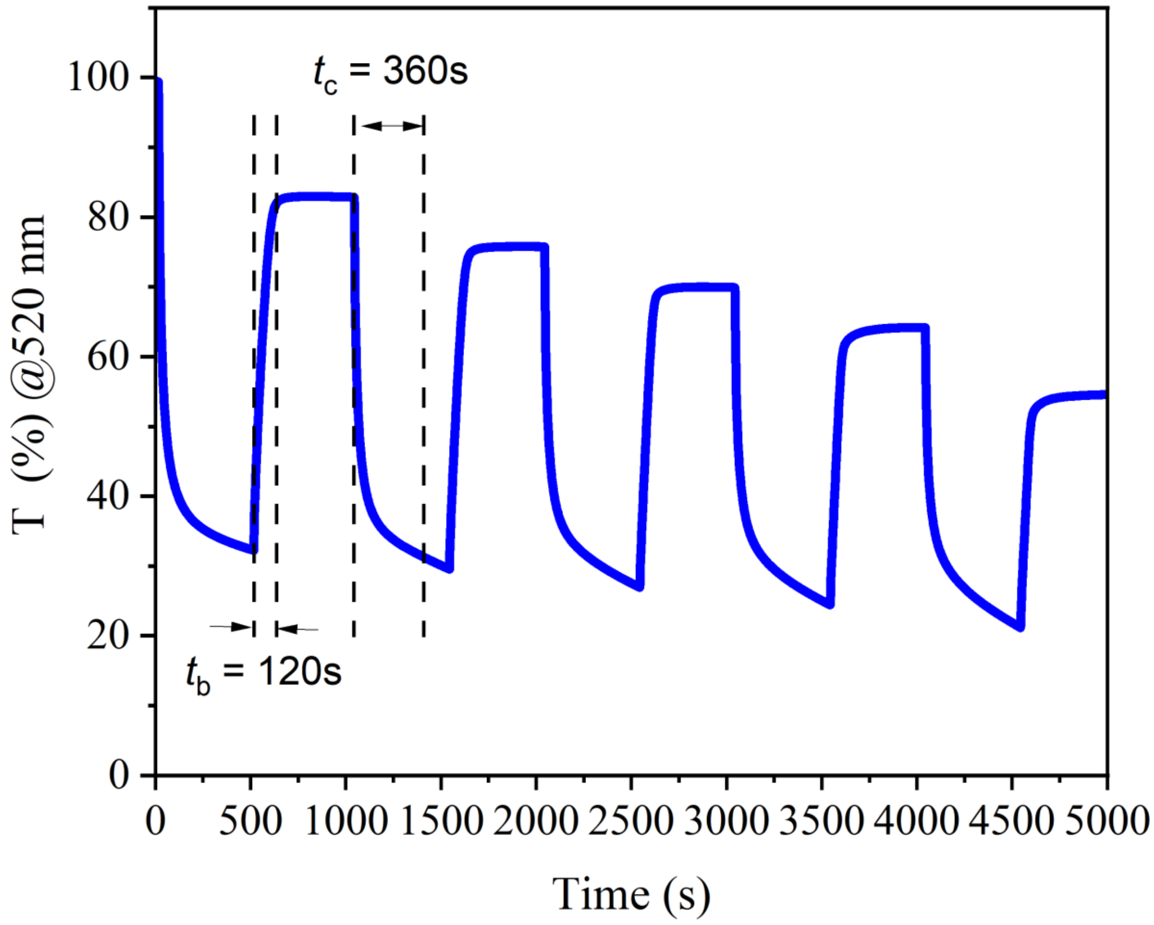

Figure S13. Electrochromic switching times and stability of electrochromic device based on complex **1**($(ClO_4)_2$).

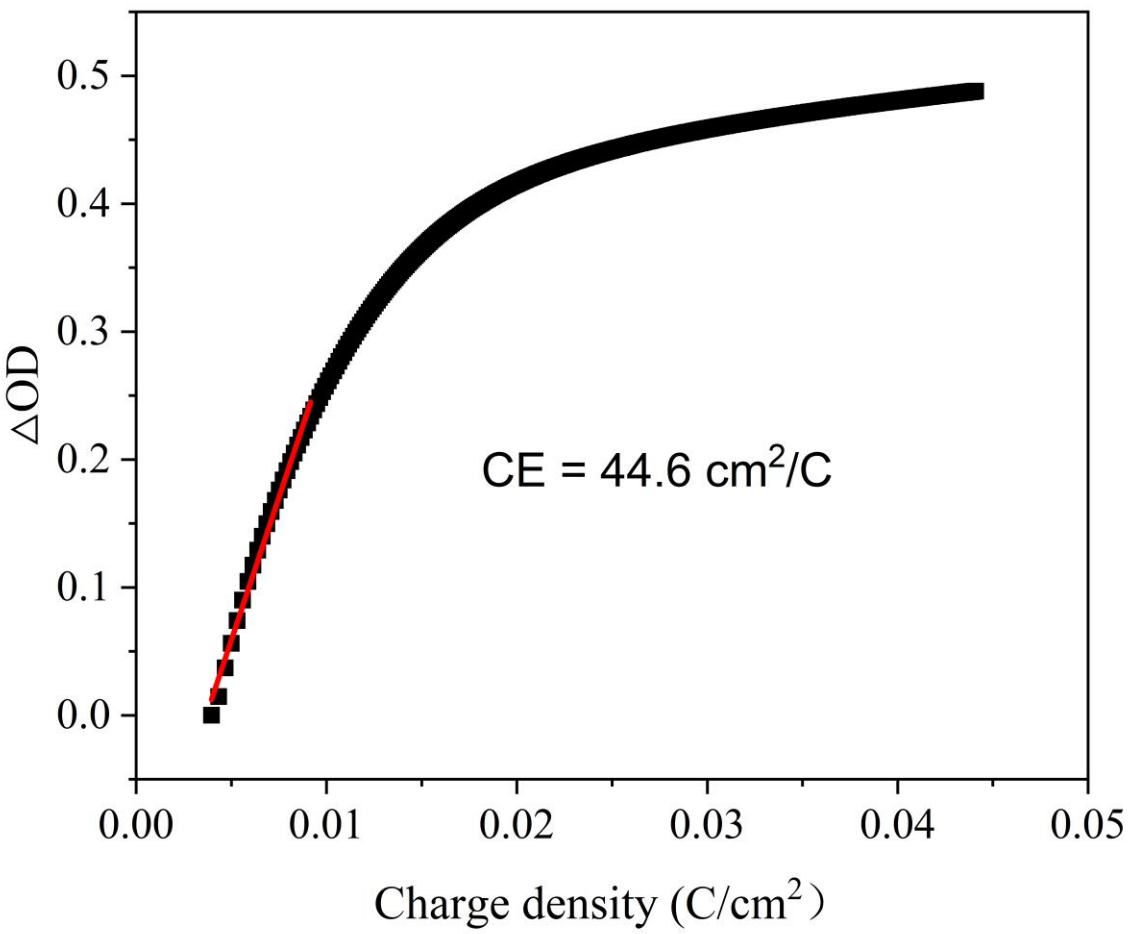

Figure S14. Plot of the optical density (ΔOD) versus the charge density (C/cm^2) of electrochromic device based on complex **1**(ClO_4)$_2$.

References

1. Gaussian 16, Revision A.03, M. J. Frisch, G. W. Trucks, H. B. Schlegel, G. E. Scuseria, M. A. Robb, J. R. Cheeseman, G. Scalmani, V. Barone, G. A. Petersson, H. Nakatsuji, X. Li, M. Caricato, A. V. Marenich, J. Bloino, B. G. Janesko, R. Gomperts, B. Mennucci, H. P. Hratchian, J. V. Ortiz, A. F. Izmaylov, J. L. Sonnenberg, D. Williams-Young, F. Ding, F. Lipparini, F. Egidi, J. Goings, B. Peng, A. Petrone, T. Henderson, D. Ranasinghe, V. G. Zakrzewski, J. Gao, N. Rega, G. Zheng, W. Liang, M. Hada, M. Ehara, K. Toyota, R. Fukuda, J. Hasegawa, M. Ishida, T. Nakajima, Y. Honda, O. Kitao, H. Nakai, T. Vreven, K. Throssell, J. A. Montgomery, Jr., J. E. Peralta, F. Ogliaro, M. J. Bearpark, J. J. Heyd, E. N. Brothers, K. N. Kudin, V. N. Staroverov, T. A. Keith, R. Kobayashi, J. Normand, K. Raghavachari, A. P. Rendell, J. C. Burant, S. S. Iyengar, J. Tomasi, M. Cossi, J. M. Millam, M. Klene, C. Adamo, R. Cammi, J. W. Ochterski,

R. L. Martin, K. Morokuma, O. Farkas, J. B. Foresman, and D. J. Fox, Gaussian, Inc., Wallingford CT, 2016.

2. A. D. Becke, Density-functional exchange-energy approximation with correct asymptotic behavior. *Phys. Rev. A* **1988**, *38*, 3098-3100.

3. C. T. Lee, W. T. Yang, R. G. Parr, Development of the Colle-Salvetti correlation-energy formula into a functional of the electron density. *Phys. Rev. B* **1988**, *37*, 785-789.

4. M. E. Casida, C. Jamorski, K. C. Casida, D. R. Salahub. Molecular excitation energies to high-lying bound states from time-dependent density-functional response theory: Characterization and correction of the time-dependent local density approximation ionization threshold. *J. Chem.Phys*. **1998**, *108*, 4439-4449.

5. R. E. Stratmann, G. E. Scuseria, M. J. Frisch. An efficient implementation of time-dependent density-functional theory for the calculation of excitation energies of large molecules. *J. Chem Phys*. **1998**, *109*, 8218-8224.

6. V. Barone, M. Cossi, J. Tomasi. A new definition of cavities for the computation of solvation free energies by the polarizable continuum model. *J. Chem. Phys*. **1997**, *107*, 3210-3221.

7. M. Cossi, G. Scalmani, N. Rega, V. Barone. New developments in the polarizable continuum model for quantum mechanical and classical calculations on molecules in solution. *J. Chem. Phys.* **2002**, *117*, 43-54.

8. D. Andrae, U. Häussermann, M. Dolg, H. Stoll, H. Preuss. Energy-adjustedab initio pseudopotentials for the second and third row transition elements. *Theor. Chim. Acta* **1990**, *77*, 123-141.

9. T. Lu, F. Chen. Multiwfn: a multifunctional wavefunction analyzer. *J. Comput. Chem.* **2012**, *33*, 580-592.
